# Opinion: To assess marine cloud brightening's technical feasibility, we need to know what to study—and when to stop

**DOI:** 10.1073/pnas.2118379119

**Published:** 2022-01-19

**Authors:** Michael S. Diamond, Andrew Gettelman, Matthew D. Lebsock, Allison McComiskey, Lynn M. Russell, Robert Wood, Graham Feingold

**Affiliations:** ^a^Cooperative Institute for Research in Environmental Sciences (CIRES), University of Colorado, Boulder, CO 80309;; ^b^Chemical Sciences Laboratory (CSL), National Oceanic and Atmospheric Administration (NOAA), Boulder, CO 80305;; ^c^Climate and Global Dynamics Laboratory, National Center for Atmospheric Research, Boulder, CO 80301;; ^d^Jet Propulsion Laboratory, California Institute of Technology, Pasadena, CA 91109;; ^e^Environmental and Climate Sciences Department, Brookhaven National Laboratory, Upton, NY 11973;; ^f^Scripps Institution of Oceanography, University of California San Diego, La Jolla, CA 92037;; ^g^Department of Atmospheric Sciences, University of Washington, Seattle, WA 98195

To avoid the worst impacts of climate change, it’s paramount that we decarbonize the economy and preserve and restore natural ecosystems. Unfortunately, pledges made from countries thus far will not limit warming to below 1.5 °C, even after accounting for the more ambitious targets set at the recent COP26 climate summit in Glasgow (1). Meeting the 1.5 °C Paris Agreement goal will likely require a massive deployment of CO_2_ removal technologies that remain unproven at scale (2). As a result, many scientists—including an expert panel recently convened by the US National Academies of Science, Engineering, and Medicine (NASEM)—have advocated for research into solar climate interventions that would offset some effects of greenhouse gas-driven warming by reflecting more sunlight back to space (3). This would temporarily cool the Earth, giving mitigation and adaptation efforts more time to scale up. One such approach, marine cloud brightening (MCB), would seed low-altitude clouds over the ocean with salt particles to produce more and smaller cloud droplets, leading to brighter clouds (4, 5).

**Figure:**
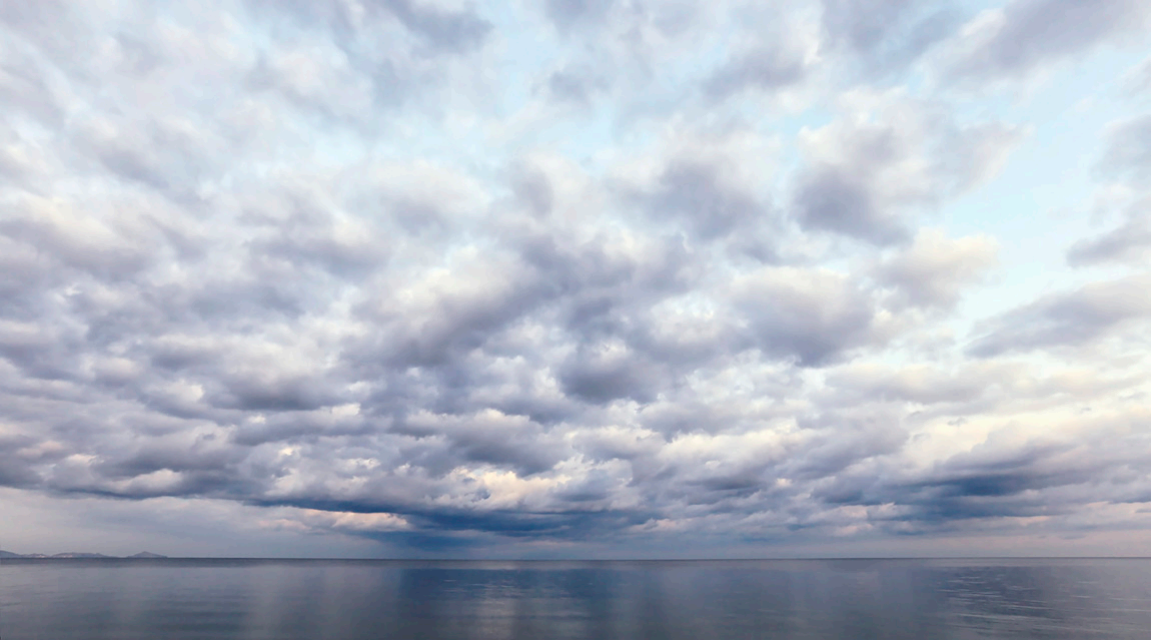
Right now, research into climate interventions such as marine cloud brightening lacks a comprehensive framework to produce and assess the information needed for sound decision making. Image credit: Shutterstock/Venera Salman.

**Figure:**
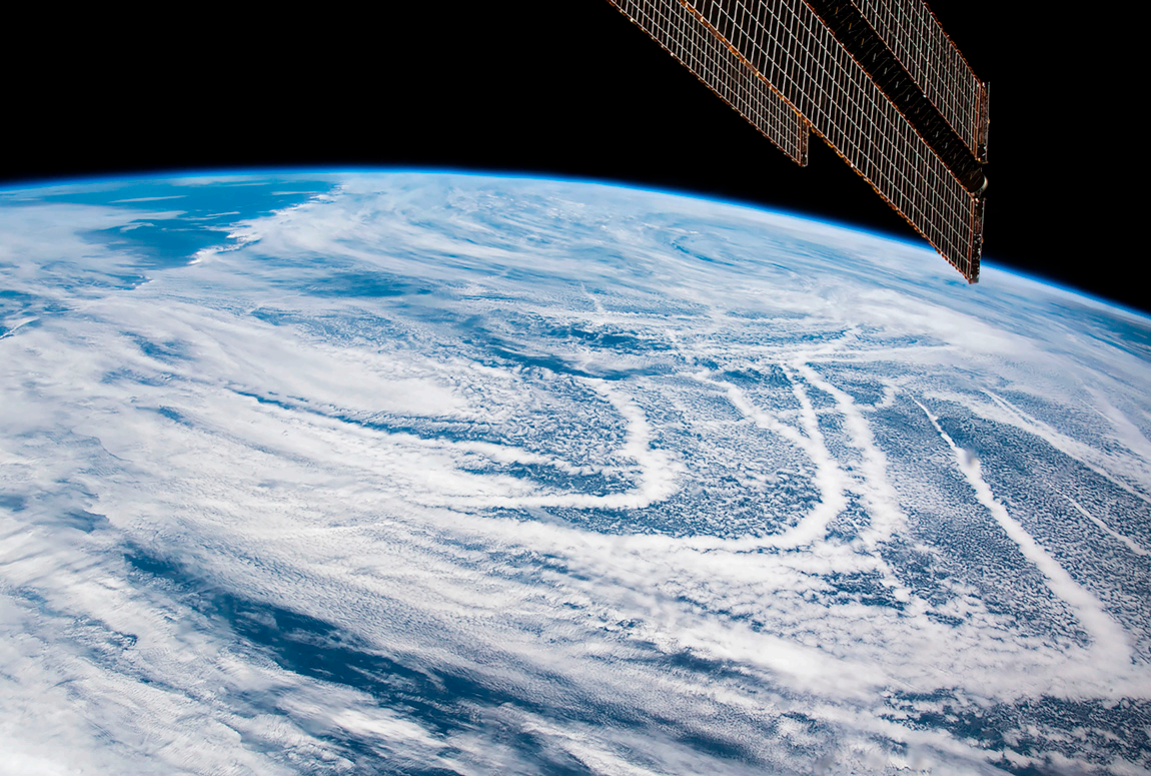
A view of marine clouds as seen from the International Space Station. Bands of brighter clouds are created by the pollution from ships' smokestacks. Marine cloud brightening proposals would aim to replicate this effect using sea salt. Astronaut photograph ISS059-E-36734. Image credit: Earth Science and Remote Sensing Unit, NASA Johnson Space Center, and NASA Earth Observatory.

We endorse the need for a transdisciplinary research program focused on MCB and other solar climate interventions. However, climate intervention research currently lacks a comprehensive framework to objectively produce and assess the information needed for sound decision-making. This is attributable in part to a lack of formal national or international research programs and governance. Without such a framework, future research investments are at risk of being unbalanced or misguided and thus failing to efficiently produce and disseminate policy-relevant knowledge in a timely manner. When considering the feasibility of a given climate intervention, we need to determine the key questions that require the most work (i.e., What should researchers study?). And we need criteria for terminating research on non-viable proposals (i.e., When should we stop?).

Our goal here is twofold. First, we provide a framework to help organize and prioritize future research on MCB. We propose that there exist six physical science “checkpoints” that underpin the technical feasibility of MCB, all of which must be addressed for MCB to be a viable option within the broader portfolio of societal responses to climate change. We define a “checkpoint” as an important, relatively self-contained topical area with open questions that must be resolved for a solar climate intervention method to be considered viable. Although we focus on the case of MCB, similar exercises will be equally important to assess the viability of other intervention proposals, such as stratospheric aerosol injection, but with different checkpoints tailored to each intervention.

Second, we maintain that the identification and broad acceptance of “exit ramps” is a first step in the direction of internal research governance to allay reasonable concerns that any climate intervention program (not just MCB) may become inappropriately “locked-in” [e.g., through institutional inertia and the creation of stakeholders invested in the continuation of such a program (6, 7)]. Following Recommendation 4.1 in the NASEM report (ref. 3), “exit ramps” refer to criteria and protocols for terminating a research program once it has been determined that a proposed intervention would not be technically or socially feasible.

Our focus on internal governance is not meant to discount the important role that more formal but slower-moving governance structures would play in an ideal world; rather, it is a recognition that the research community needs self-directed measures now. MCB research is already proceeding, and given the stakes, we cannot afford to indefinitely delay research until formal structures are in place. Furthermore, progress from the research community can aid in the development of formal national research programs, and the development of an international program will be aided by national programs (see, e.g., Chapter 5 of ref. 3).

## Checkpoints and Exit Ramps

**Fig. 1.**
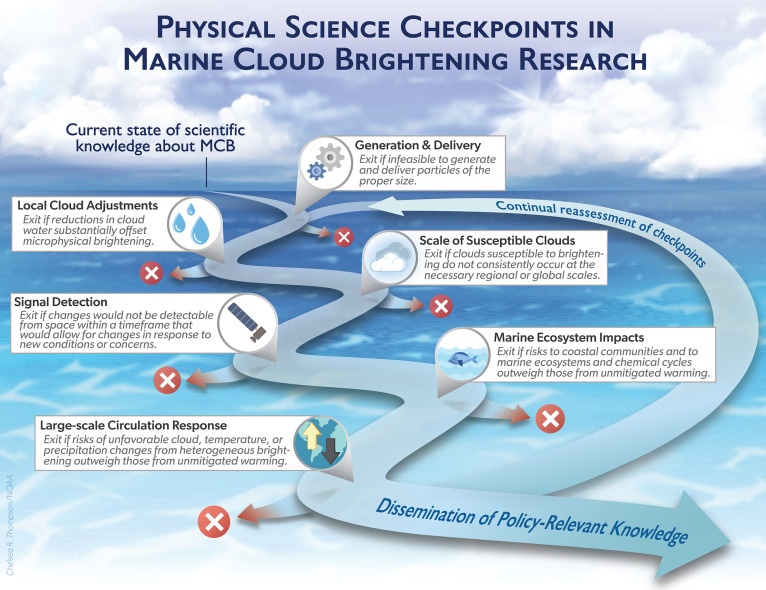
Research should proceed simultaneously on all six proposed physical science checkpoints, illustrated here, and on social science and ethics checkpoints. The checkpoints are subject to continual reassessment as research progresses. Image credit: Michael Diamond and Chelsea Thompson (CIRES and NOAA CSL, Boulder, CO).

Fig. 1 shows a schematic of our framework as a path in which each checkpoint detailed below is associated with a potential exit ramp. Research continues as long as all checkpoints are passed, but if the research and policymaking communities decide to take any exit ramp, the entire research program would stop. The checkpoints are not sequential—indeed, research on all fronts, including social science and ethical aspects of feasibility, should proceed simultaneously—and are only presented as such for the sake of illustration.

Decisions regarding these “physical science” checkpoints cannot be made by physical scientists alone. A full assessment of each checkpoint may also require expertise in technology, ecology, the social sciences, and ethics as well as engagement with policymakers and relevant stakeholders. Determining what would constitute a threshold for the magnitude of unresolved uncertainty or of potential Earth system or societal responses that should trigger an exit ramp—and when an exit ramp is actually triggered—will require a broader discussion amongst the physical science community, as well as other relevant disciplines, policymakers, and stakeholders.

**Fig. 2.**
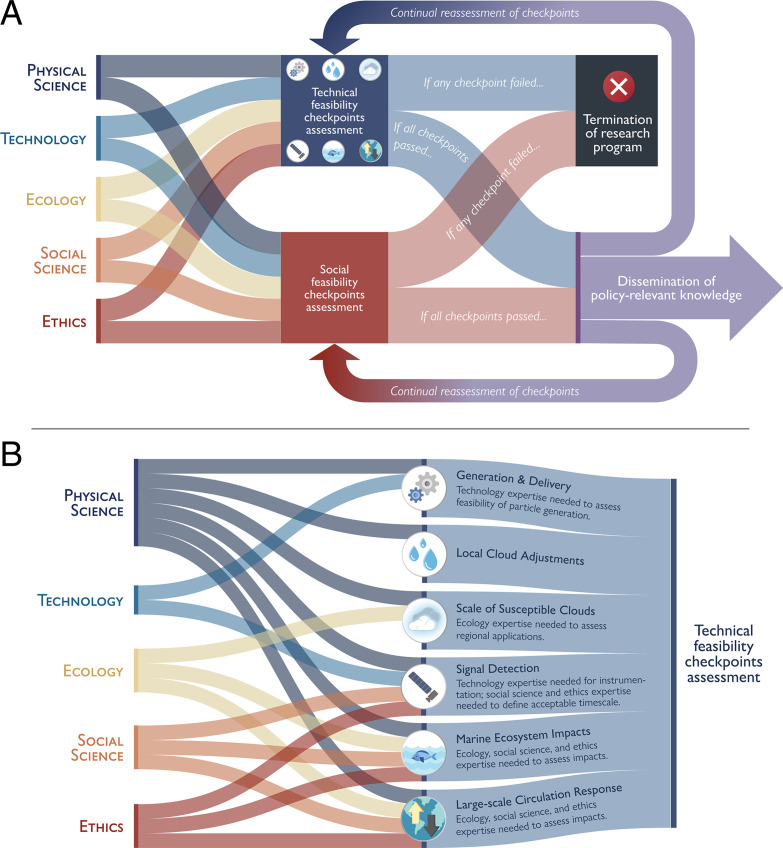
Information flows will inform the assessment of technical and social feasibility checkpoints and thus decisions on whether to take an exit ramp or to continue research. In (*A*), we show flows of information from five disciplines to the assessment of technical and social feasibility checkpoints. In (*B*), we depict the information flows most relevant to decision-making on exit ramps pertaining to each of the six proposed physical science checkpoints for the technical feasibility of MCB. Other disciplinary connections may be appropriate as research progresses. Image credit: Michael Diamond and Chelsea Thompson (CIRES and NOAA CSL, Boulder, CO).

Fig. 2 illustrates this concept as flows of information from multiple disciplines that feed into the assessment of technical and social feasibility checkpoints. Passage of the checkpoints would be subject to continual reassessment as research progresses. Lessons learned in the course of research will also inform the refinement of exit ramp criteria and potential identification of additional checkpoints and exit ramps.

Social feasibility checkpoints and exit ramps (e.g., regarding the fiscal and opportunity costs of research) can also be identified and deserve equal attention. Although the present piece focuses on the physical sciences (based on the authors' areas of expertise), all areas must progress in parallel, and in collaboration, to provide evidence-based information on the physical, technological, ecological, social, and ethical aspects of these decisions.

## Technical Feasibility of MCB

We identify six different critical science areas (checkpoints) where satisfactory answers are needed for MCB to be deemed technically feasible. The order of the checkpoints loosely corresponds to the spatiotemporal scales involved and the degree of transdisciplinarity required to answer key questions. All checkpoints are equally important, as the failure of any one would trigger an exit ramp terminating research on all.

### Generation and delivery of appropriately sized particles.

It must be shown that generators can be designed to produce aerosols (airborne particles) of an appropriate size and number that can be delivered to the marine cloud base and activate to form cloud droplets that are effective at scattering solar radiation. Not only must the particles be produced with a specific size and minimal variation, but they must also overcome any initial decrease in buoyancy owing to evaporation and not change their size distribution in an adverse manner as a result of microphysical processes while en route to the cloud base. Tackling this problem requires advances in technology and in the physical science underpinning the selection of the desired aerosol size distributions. Controlled indoor and eventually small-scale outdoor experimentation with deliberate particle release would be necessary to confidently conclude that the generation and delivery of appropriately sized particles is feasible under a range of conditions.

### Local cloud adjustments.

Aerosol perturbations must not generate cloud system responses (adjustments) that substantially offset the instantaneous brightening effect. Particles that are too small or too numerous may dissipate clouds through enhanced evaporation, whereas particles that are too large may dissipate clouds through enhanced precipitation (8). Responses in the form of local secondary circulations might also offset a portion of the brightening (9). For MCB to be effective, reductions in cloud water attributable to enhanced evaporation or precipitation or secondary circulations would have to be sufficiently small relative to the brightening effects derived from smaller cloud droplet sizes and reduced drizzle in already precipitating clouds. The nature of the cloud and radiation responses to an aerosol perturbation needs to be understood for a range of potential cloud and weather states. In situ and remote sensing measurements from small-scale outdoor experimentation (one to several salt tracks) would be invaluable for challenging high-resolution cloud models in an assessment of our ability to predict adjustments under various conditions.

### Spatiotemporal scale of susceptible clouds.

Clouds that have the potential to be brightened by aerosol injections (susceptible clouds) must exist consistently on a sufficient spatiotemporal scale for MCB to have a global impact on Earth’s energy budget and thus surface temperature. Cloud susceptibility varies with weather, which will vary significantly in a practical MCB setting. It is thus essential that we account for meteorological conditions in our assessment of the integrated effects of MCB. Knowledge of the local cloud response under different weather conditions and the frequency and extent of occurrence of those weather states would allow extrapolation to the global scale of susceptible clouds.

A caveat here is that even if susceptible clouds do not exist at a large enough scale to have a global impact, they may exist in certain regions with particularly heat-sensitive ecosystems, such as coral reefs (10). In this case, regionally limited proposals may still be viable and merit a continued, albeit diminished, research program.

### Signal detection.

The radiative effect of MCB must be observable to demonstrate that the intervention is working as intended. This requires demonstration that it is possible to observe metrics of brightening from space with current and projected technology, within a timescale sufficient to allow for adjustments in response to new conditions or concerns. Unfortunately, the relatively small signals anticipated by MCB activities may require timeframes on the order of years to decades for statistically significant detection (11, 12). Determining acceptable timescales, and in particular addressing the question of the point at which extended tests of MCB essentially become outright deployment (13), will require collaborating with the social science and ethics research communities as well as relevant policymakers.

### Impacts on marine ecosystems and coastal communities.

In current MCB proposals, the injected aerosols would be sea salt. Although recent findings suggest that perturbations to the existing salt burden from MCB could be quite small (14), a viable MCB activity must show that the addition of salt (and therefore chloride) to the marine boundary layer would not significantly interfere with important chemical processes or have unacceptably negative impacts on coastal communities and ecosystems. An increase in salt aerosol may have the beneficial effect of reducing tropospheric ozone pollution (15), although the evidence for the chemical effects of MCB is currently very limited and important aspects of marine chemical cycles remain poorly constrained (16). Changes in, for example, the total amount of downwelling sunlight and the ratio of direct to diffuse light may affect ecosystems around MCB deployments in largely unknown ways (17). Salt deposition may also affect ecosystem functions as well as coastal infrastructure.

To fully understand the potential benefits and dangers of MCB in context, the risks to ecosystems and coastal communities from climate intervention would need to be assessed in light of the risks from unmitigated warming. Collaboration between physical scientists, ecologists, social scientists, and ethicists will be necessary to both identify and answer the most pressing questions regarding potential impacts on marine ecosystems and coastal communities (18).

### Large-scale circulation and precipitation response.

Spatially heterogeneous cloud brightening could lead to large-scale circulation responses with unintended consequences, such as darkening clouds elsewhere (thus being counterproductive) or affecting precipitation patterns in vulnerable regions like the Amazon (19–21). For MCB to be viable, unacceptably deleterious remote effects via large-scale circulation changes must be highly unlikely. As with the previous checkpoint, assessing the risks of MCB also requires an understanding of the risks from unmitigated warming and its resulting global and regional circulation and precipitation changes. Defining exactly which regions are “vulnerable” and thus of greatest concern and what level of risk would be “unacceptable” will require substantial contributions from the fields of ecology, social science, and ethics as well as from potentially affected communities.

Assessment of large-scale responses is particularly challenging because it requires an understanding of multiple atmospheric processes (microphysical, dynamical, and radiative) occurring at scales ranging from microns and seconds to thousands of kilometers and decades. Numerical models, our only tool for assessing these cross-scale interactions, are not yet up to the task. They will require substantial improvements in their representation of cloud and related aerosol and chemical processes, and in computational resources, before we have a more robust understanding of the predictability of these responses.

## The Path Ahead

Although existing research has begun to address all of these themes, the road past these checkpoints is still long and arduous. Although our discussion of next steps below is focused primarily on research activities within the United States, where all the authors currently reside and work, international cooperation, collaboration, and data sharing will be necessary components of the climate intervention research enterprise. In particular, adequately resourced partnerships, exchanges, and other capacity-building measures with nations and communities traditionally underserved by the global research enterprise will be critical for climate intervention research to achieve legitimacy.

Addressing the physical science checkpoints identified here will require substantial work, including the development of new modeling and observational methods that encompass a wide range of scales and an international commitment to providing accurate space-based measurements into the future. Fortunately, planned or proposed investments in atmospheric research hold promise for developing the tools we need to approach MCB decision-making thoughtfully, at least from the physical science side. New satellite missions carrying state-of-the-art instrumentation for aerosol–cloud retrievals and radiative signal detection will augment more focused aircraft in situ measurements in regions of interest (22, 23). A proposed large-scale aerosol–cloud-turbulence laboratory facility could improve our fundamental understanding of cloud microphysics (24). A fusion of observations, experiments, and numerical models will greatly improve our ability to understand and predict responses in the physical system at the scales necessary for addressing the technical and social feasibility of MCB. These investments are equally needed to monitor how human activities are already altering clouds through emission of pollutants and for monitoring the climate more generally.

A responsible and responsive MCB research program must ensure that all technical and social feasibility checkpoints are addressed, because the decision to take any exit ramp necessarily means that further MCB-specific research activities would cease. Successful passage of the physical science checkpoints identified here would boost confidence in the potential for MCB to help cool the planet, provided that we can also overcome the formidable obstacles to its technological and societal viability.
